# Classical Bovine Spongiform Encephalopathy by Transmission of H-Type Prion in Homologous Prion Protein Context

**DOI:** 10.3201/eid1709.101403

**Published:** 2011-09

**Authors:** Juan-María Torres, Olivier Andréoletti, Caroline Lacroux, Irene Prieto, Patricia Lorenzo, Magdalena Larska, Thierry Baron, Juan-Carlos Espinosa

**Affiliations:** Author affiliations: Centro de Investigación en Sanidad Animal, Madrid, Spain (J.-M. Torres, I. Prieto, P. Lorenzo, M. Larska, J.-C. Espinosa);; Ecole Nationale Vétérinaire de Toulouse, Toulouse, France (O. Andréoletti, C. Lacroux);; Agence Francaise de Sécurité Sanitaire des Aliments, Lyon, France (T. Baron)

**Keywords:** bovine spongiform encephalopathy, atypical BSE, H-type BSE, BSE, origin of BSE, prion strain, prion transmission, PrP, prions and related diseases, research

## Abstract

Bovine spongiform encephalopathy (BSE) and BSE-related disorders have been associated with a single major prion strain. Recently, 2 atypical, presumably sporadic forms of BSE have been associated with 2 distinct prion strains that are characterized mainly by distinct Western blot profiles of abnormal protease-resistant prion protein (PrP^res^), named high-type (BSE-H) and low-type (BSE-L), that also differed from classical BSE. We characterized 5 atypical BSE-H isolates by analyzing their molecular and neuropathologic properties during transmission in transgenic mice expressing homologous bovine prion protein. Unexpectedly, in several inoculated animals, strain features emerged that were highly similar to those of classical BSE agent. These findings demonstrate the capability of an atypical bovine prion to acquire classical BSE–like properties during propagation in a homologous bovine prion protein context and support the view that the epidemic BSE agent could have originated from such a cattle prion.

## Abstract 2

TOC Summary: An epidemic agent could have originated from such a cattle prion.

Transmissible spongiform encephalopathies, or prion diseases, are a group of neurodegenerative disorders that include Creutzfeldt-Jakob disease (CJD) in humans, scrapie in sheep and goats, and bovine spongiform encephalopaty (BSE) in cattle. Prion diseases are characterized by specific histopathologic lesions and deposits of an abnormal conformational isoform (PrP^Sc^) of the host-encoded physiologic prion protein (PrP^C^) in the central nervous system. PrP^Sc^ but not PrP^C^ is partially resistant to digestion by proteinase K, resulting in an N terminally truncated prion protein termed PrP^res^ that can be detected by Western blot and showing a characteristic banding pattern that reflects the 3 PrP^res^ glycoforms. The apparent molecular masses and relative quantities of these glycoforms are used in biochemical PrP^res^ typing as the criteria to differentiate between prion diseases.

BSE is a prion epidemic that has caused the deaths of ≈200,000 cattle in Europe, mainly in the United Kingdom, since it emerged in 1985. Although multiple agent strains have been identified in sheep scrapie (*1**,**2*) and human CJD (*3**,**4*), early evidence showed that BSE was caused by a single major strain (*5**,**6*) with the ability to efficiently cross the species barriers and showing stable features even when transmitted to other species. Transmission of BSE to humans through contaminated food is believed to be responsible for variant CJD (vCJD) (*7**,**8*). Several authors reported that BSE and vCJD prions share similar strain-specific features, including a unique PrP^res^ molecular signature (*6**,**9**,**10*), after transmission to mice or macaques. However, other studies described the production of different PrP^res^ molecular signature after BSE and vCJD prions transmission in wild-type (*11*) and human PrP transgenic mice (*12*,*13*).

Epidemiologic investigations identified contaminated meat and bone meal as the vehicle that recycled the BSE agent in the cattle population (*14*). However, the origin of BSE remains under debate, and the disease has been hypothesized to have derived either from sheep scrapie or from a spontaneous bovine prion disease analogous to sporadic forms of CJD in human (*15*) or even from human transmissible spongiform encephalopathy (*16*).

More recently, 2 atypical forms of BSE have been identified in several European countries (*17*), Japan (*18**,**19*), the United States (*20*), and Canada (*21*). Several studies suggest that these atypical disorders are associated with 2 distinct prion strains that are mainly characterized by distinct PrP^res^ profiles, named high-type (H-type) and low-type (L-type) according to the electrophoretic migration of the unglycosylated PrP^res^, which is higher (BSE-H) or lower (BSE-L) than classical BSE (BSE-C) (*22*). An additional distinctive signature of H-type and L-type PrP^res^ is the smaller proportion of the diglycosylated PrP^res^ compared with the classical-type (C-type) PrP^res^, more obvious in L-type BSE (*23**–**25*).

All epidemiologic and biologic evidence strongly suggests that BSE-H and BSE-L represent sporadic forms of BSE (*23**,**24*) associated with 2 distinct prion strains. Transmission experiments in different mouse models, including transgenic mice expressing bovine PrP, showed that BSE-H and BSE-L exhibited strain-specific features clearly distinct between each other that also differed from BSE-C (*13**,**25**–**28*). However, BSE-L isolates unexpectedly showed transmission of a disease with some phenotypic features that resembled those of the BSE-C agent when inoculated in either transgenic mice expressing ovine PrP (*28*) or inbred wild-type mouse lines (*25*), suggesting that atypical bovine strains can modify their properties, at least after species barrier passages, converging with those of BSE-C.

We show that the transmission of atypical BSE-H isolates in transgenic mice expressing homologous bovine prion protein (PrP) led to emergence of a clearly distinct prion with strain features similar to those of the BSE-C agent and that such similarities were maintained on subsequent passages. These observations provide new insights into the nature of the events that could have led to the BSE epizootic.

## Materials and Methods

### Transgenic Mice

We used *Tg110* transgenic mice in all inoculation experiments. This mouse line expresses bovine PrP^C^ (≈8× that of the level of PrP^C^ in cattle brain) under the control of the mouse *prnp* gene promoter in a mouse PrP^0/0^ background (*29*).

### BSE Isolates

The 5 BSE-H isolates used in this study comprised brainstem samples from naturally affected cows, diagnosed as atypical H-type BSE on the basis of the molecular analyses of PrP^res^ (*23*). All cows were healthy and killed at 8–15 years of age. Four (isolates 07-644, 03-440, 03-2095, and 02-2695) were provided by the Agence Française de Sécurité Sanitaire des Aliments (Lyon, France). Isolate 45 was obtained from the Polish National Veterinary Research Institute (Pulawy, Poland). For comparative studies, material obtained from brainstem of 1 cow naturally infected with BSE-C (RQ 225:PG1199/00), supplied by the Veterinary Laboratories Agency (New Haw, Addlestone, Surrey, UK), was used as BSE-C control. For mouse inoculations, all isolates were prepared from brain tissues as 10% (wt/vol) homogenates. For subpassages, 10% brain homogenates from *Tg110* mice collected from primary passage were used as inocula.

All inocula were prepared in sterile 5% glucose as 10% homogenates. Each inoculum was prepared separately in a biosafety cabinet according to a strict protocol to avoid cross-contamination. To diminish the risk for bacterial infection, the inocula were preheated for 10 min at 70°C before inoculation.

### Mouse Transmission Studies

Groups of 6–12 mice (6–7 weeks of age, weighing ≈20 g) were inoculated with 20 µL of the appropriate sample in the right parietal lobe by using 25-gauge disposable hypodermic syringes. UNO MICRO ID-8 ISO transponders (Roestvaststaal BV, Zevenaar, the Netherlands) were used for individual identification of mice. After inoculation, mice were observed daily and their neurologic status were assessed 2×/wk. When progression of the disease was evident, animals were euthanized. All animals were housed in accordance with guidelines of the Code for Methods and Welfare Considerations in Behavioral Research with Animals of the European Union directive 86/609EC). Necropsy was performed, and brain and spleen were taken. Part of the sample was frozen at −20°C for biochemical analysis, and the remaining part was fixed for histopathologic studies.

Survival times were calculated for each inoculum as the time between inoculation and euthanasia in days and expressed as the mean of the survival days postinoculation (dpi) of all the inoculated mice with its correspondent standard error of the mean. Data were processed by using SigmaPlot 2001 software (Systat Software, San Jose, CA, USA).

### PrP^res^ Western Blotting

Frozen mouse brain samples were prepared as 10% (wt/vol) homogenates in 5% glucose in distilled water in grinding tubes (Bio-Rad, Hercules, CA, USA) by using a TeSeE Precess 48 homogenizer (Bio-Rad) following the manufacturer’s instructions. All samples were analyzed by Western blot by using the kit TeSeE Western Blot 355 1169 (Bio-Rad) but with some adjustments for the different amounts of samples used. To achieve the volume proposed in the manufacturer’s recommendations, 100 μL of the brain homogenates to be tested were supplemented with 100 μL of a 10% brain homogenate from PrP null mice (*30*). Processed samples were loaded on Criterion 12% polyacrylamide gels from Bio-Rad (165.6001) and electrotransferred to immobilon membranes (IPVH 000 10 [Millipore, Billerica, MA, USA]). For the immunoblotting experiments, Sha31 (*31*), Saf84 (Cayman Chemical, Ann Arbor, MI, USA) and 12B2 (*32*) monoclonal antibodies (mAbs) were used at a concentration of 1 μg/mL. Sha31 recognizes _156_YEDRYYRE_163_ epitope, and Saf84 recognizes _171_QVYYRPVDQYS_181_ epitope and 12B2 recognizes _101_WGQGG_105_ epitope of the bovine PrP sequence. Immunocomplexes were detected by horseradish peroxidase–conjugated antimouse immunoglobulin G (Amersham Pharmacia Biotech, Piscataway, NJ, USA). Immunoreactivity was visualized by chemiluminescence (Amersham Pharmacia Biotech) and obtained after exposition with medical radiographic film (Agfa, Mortsel, Belgium).

### Histopathologic Analysis

All procedures involving mouse brains and spleens were performed as described (*33*). Briefly, samples were fixed in neutral-buffered 10% formalin (4% formaldehyde) before embedding in paraffin. Once deparaffinized, 2-µm–thick tissue sections were stained with hematoxylin and eosin. Lesion profiles of the brains were established according to the standard method described by Fraser and Dickinson (*34*). For paraffin-embedded tissue blots, the protocol described by Andréoletti et al. (*35*) was used.

## Results

**Figure 1:**
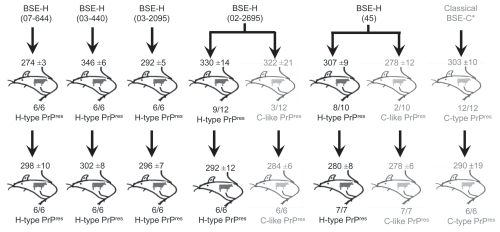
Overview of transmission of BSE-H isolates in *tg110* mice. Five different isolates were intracerebrally inoculated into groups of 6–12 mice per isolate. Survival times at different serial passages are indicated as mean ± SD days postinoculation. Molecular profiles exhibited in the brains of inoculated mice are indicated as H-type, C-type, or C-like PrP^res^, and proportion of mice showing each profile. Previously reported data on BSE-C transmission in these mice (*36*) are included here only for comparison. BSE, bovine spongiform encephalitis; BSE-H, unglycosylated PrP^res^ that is higher than BSE-C; H-type, high-type Western blot profile of PrP^res^; C-type, classical-type Western blot profile of PrP^res^; C-like, classical BSE–like; PrP^res^, protease-resistant prion protein; BSE-C, classical BSE.

The transmission dynamic of BSE-H agent into *Tg110* mice is similar to that of BSE-C. The 4 BSE-H isolates from France and 1 from Poland that were intracerebrally inoculated into transgenic mice expressing bovine PrP (*Tg110* mice) induced a typical neurologic disease on primary transmission, with a 100% attack rate (Figure 1). Remarkably, the survival times (mean ± SD 274 ± 3 to 346 ± 6 dpi) were similar than those produced by several BSE-C isolates (≈300 days) on the same *Tg110* mouse line (*29**,**36**,**37*). The longest mean survival times observed for mice infected with isolates 03–440 (mean ±SD 346 ±6 dpi) and 02–2695 (330 ±14 dpi) could reflect a lower infectivity of these isolates, consistent with its comparatively lower PrP^res^ content (data not shown). Moreover, the survival time of mice infected with these 2 isolates was reduced on subpassage, approaching that for BSE-C or BSE-H isolates of presumably higher titer (i.e., producing no substantial reduction of survival time on subpassage: isolates 07-644, 03-2095, and 45).

### PrP^res^ Molecular Profiles of BSE-H–Inoculated *Tg110* Mice

**Figure 2:**
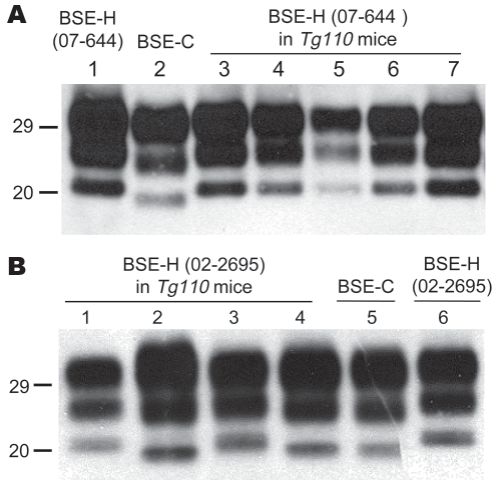
Western blot analyses of brain protease-resistant prion protein (PrP^res^) from BSE-H infected mice by using Sha31 monoclonal antibody. A) Mice infected with isolate 07-644 at first passage (lanes 3–7) showing a homogeneous high-type (H-type) PrP^res^ molecular profile; BSE-H isolate 07-644 (lane 1) and a BSE-C isolate (lane 2) were included for comparison. B) Mice infected with isolate 02-2695 at first passage showing either H-type (lanes 1 and 3) or classical BSE–like (C-like) PrP^res^ molecular profile (lanes 2 and 4); BSE-H isolate 02-2695 (lane 6) and a BSE-C isolate (lane 5) were included for comparison. In panel B, a 10-fold equivalent brain tissue mass was loaded for brains from mice showing H-type PrP^res^ molecular profile (lanes 1 and 3) than from those with a C-like PrP^res^ molecular profile (lanes 2 and 4) to obtain equivalent PrP^res^ signals. Values to the left indicate molecular mass in kDa. BSE, bovine spongiform encephalopathy; BSE-H, unglycosylated PrP^res^ that is higher than BSE-C; BSE-C, classical BSE.

The brains of inoculated mice were examined for PrP^res^ by Western blot analysis with Sha31 mAb. Consistent with the efficient transmission observed, PrP^res^ was readily detected from the first passage in all *Tg110* mice inoculated with the different BSE-H isolates. In 3 BSE-H isolates (07-644, 03-440, and 03-2095), the totality (100%) of the inoculated *Tg110* mice produced a PrP^res^ profile similar to that in cattle (H-type PrP^res^) but clearly distinct from that produced by BSE-C agent (C-type PrP^res^) in cattle (Figure 2, panel A; data not shown). Compared with C-type PrP^res^, H-type PrP^res^ was characterized by a significantly higher apparent molecular mass of the unglycosylated band. The results obtained with these 3 isolates were comparable with those obtained by other authors with 2 BSE-H isolates inoculated in a different bovine PrP mouse line (*27*), where they concluded that the H-type agent essentially retained its biochemical phenotype upon serial transmission to bovine PrP transgenic mice.

**Figure 3:**
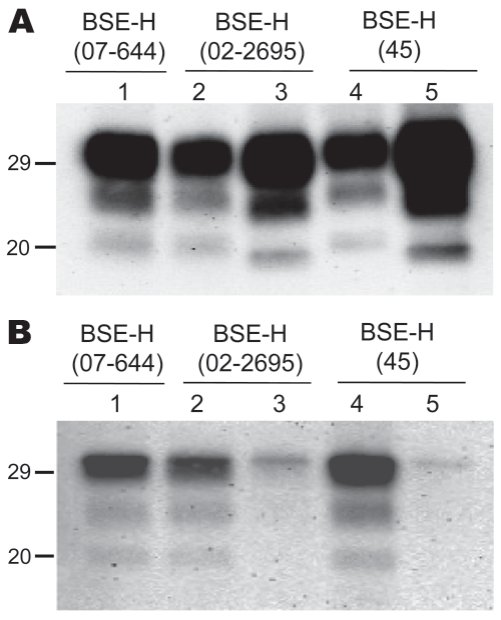
Comparative Western blot analyses with Sha31 and 12B2 monoclonal antibodies (mAbs) of brain protease-resistant prion protein (PrP^res^) from BSE-H–infected mice. Mice infected with isolate 07-644 (lane 1), 02-2695 (lanes 2 and 3), or 45 (lanes 4 and 5) at first passage showing either high-type (lanes 1, 2, and 4) or classical BSE–like PrP^res^ molecular profile (lanes 3 and 5). Panel A was shown with Sha31 mAb; panel B was shown with 12B2 mAb. The same quantities of PrP^res^ were loaded in both panels A and B. Values to the left indicate molecular mass in kDa. BSE, bovine spongiform encephalopathy; BSE-H, unglycosylated PrP^res^ that is higher than classical BSE.

**Figure 4:**
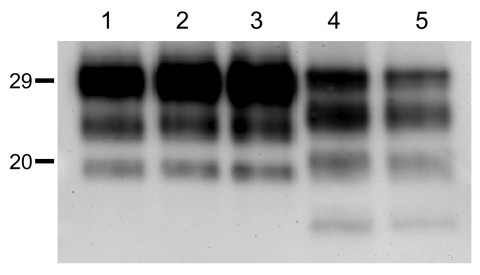
Western blot analyses of brain protease-resistant prion protein (PrP^res^) from BSE-H infected mice by using Saf84 monoclonal antibody. *Tg110* mice infected with isolate 02-2695 (lanes 2 and 3) or 45 (lane 4) at first passage showing either high-type (lane 2) or classical BSE–like PrP^res^ molecular profile (lanes 3 and 4). The BSE-H isolate (02–2695) (lane 1) and a BSE-C isolate (lane 5) were included for comparison. Similar quantities of PrP^res^ were loaded in each lane. Values to the left indicate molecular mass in kDa. BSE, bovine spongiform encephalopathy; BSE-H, unglycosylated PrP^res^ that is higher than BSE-C; BSE-C, classical BSE.

We observed a different situation for the other 2 BSE-H isolates (02-2695 from France and 45 from Poland), where 3 and 2, respectively, of infected mice (Figure 1) showed a PrP^res^ profile clearly distinct from that of BSE-H in cattle (Figure 2, panel B). These 5 mice produced a PrP^res^ with lower (≈1.5 kDa) apparent molecular mass of the 3 PrP^res^ glycoforms, which was indistinguishable from that produced by BSE-C agent in these mice (Figure 2, panel B, and data not shown). Further characterization of this PrP^res^ with other antibodies showed that the PrP^res^ produced by these mice was not recognized by 12B2 mAb (Figure 3). However, 12B2 immunoreactivity against the H-type PrP^res^ produced by other mice (inoculated at the same time with the same isolates) remains essentially similar to that in cattle BSE-H (Figure 3). Furthermore, PrP^res^ immunolabeling with Saf84 mAb showed that these mice, contrary to mice with H-type PrP^res^, did not retain the characteristic PrP^res^ band profile (4 bands) of cattle BSE-H but showed a PrP^res^ profile (3 bands) similar to that of BSE-C in *Tg110* mice (Figure 4).

**Figure 5:**
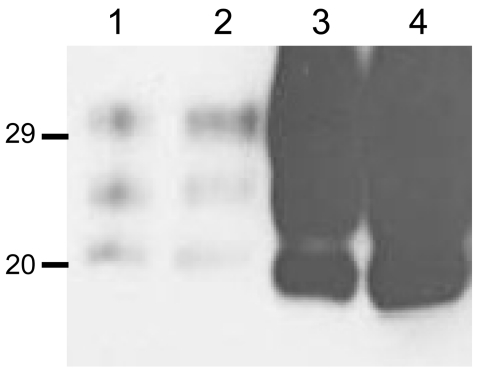
Comparison of the amount of protease-resistant prion protein (PrP^res^) in brain sample from mouse inoculated with BSE-H (isolate 02-2695) showing either high-type (lane 1 and 2, first and second passages, respectively) or classical BSE–like PrP^res^ molecular profile (lanes 3 and 4, first and second passages, respectively). Identical amounts of 10% brain homogenate were loaded in each lane. Western blot was shown with Sha31 monoclonal antibody. Values to the left indicate molecular mass in kDa. BSE, bovine spongiform encephalopathy; BSE-H, unglycosylated PrP^res^ that is higher than classical BSE–like.

In addition, the proportion of the diglycosylated PrP^res^ increased in comparison with the mice with H-type features, as shown by using Sha31 mAb (Figure 2, Figure 3). Another difference was that the PrP^res^ level in brain was much higher than that in mouse brains with H-type PrP^res^ but similar to that in mouse brains inoculated with BSE-C, as shown by comparative Western blot analysis by using Sha31 mAb (Figure 5) and by the different equivalent brain tissue masses loaded to obtain equivalent PrP^res^ signals (Figure 2, Figure 3, Figure 4). A 10-fold equivalent brain tissue mass was loaded for brains from mice showing H-type PrP^res^ molecular profile than from those with a classical-like (C-like) PrP^res^ molecular profile to obtain equivalent PrP^res^ signals (Figure 2, Figure 3, Figure 4). These mice thus showed PrP^res^ molecular features indistinguishable from those in *Tg110* mice infected with C-like features.

For these 2 isolates, a second passage was performed in *Tg110* mice by using a brain homogenate derived from a mouse with either H-type or C-like PrP^res^ (Figure 1). Survival times did not differ substantially when the different inocula were compared (H-type vs. C-like PrP^res^ brain homogenate) or when compared with second passages of BSE-C in these mice. All the mice inoculated with the H-type brain homogenates showed H-type PrP^res^ features, whereas all the mice inoculated with the C-like brain homogenates exhibited C-type PrP^res^ molecular features indistinguishable from that of BSE-C (data not shown).

### Lesion Profiles and PrP^Sc^ Deposition Patterns in BoPrP-*Tg110* Mice

**Figure 6:**
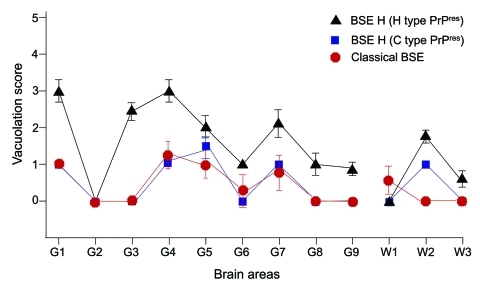
Vacuolar lesion profiles in brains from *Tg110* mice inoculated with BSE-H (isolate 02-2695, first passage) showing either H-type PrP^res^ phenotype (black triangles, n = 6 animals) or C-like PrP^res^ phenotype (blue squares, n = 3 animals). Lesion profile in brains from *Tg110* inoculated with BSE-C (first passage) is also included for comparison (red circles, n = 6 animals). Lesion scoring was undertaken for 9 areas of gray matter (G) and white matter (W) in mouse brains: dorsal medulla (G1), cerebellar cortex (G2), superior colliculus (G3), hypothalamus (G4) medial thalamus (G5), hippocampus (G6), septum (G7), medial cerebral cortex at the level of the thalamus (G8) and at the level of the septum (G9), cerebellum (W1), mesencephalic tegmentum (W2), and pyramidal tract (W3). Error bars indicate SE. BSE, bovine spongiform encephalopathy; BSE-H, unglycosylated PrP^res^ that is higher than BSE-C; PrP^res^, protease-resistant prion protein; H-type, high-type Western blot profile of PrP^res^; C-type, classical-type Western blot profile of PrP^res^; C-like, classical BSE–like; BSE-C, classical BSE.

We next examined vacuolation and PrP^Sc^ distribution in the brain, which are known to vary by strain (*10**,**34**,**38*). In general, mice brains exhibiting H-type PrP^res^ correlated with overall more intensive vacuolation that is pronounced in areas such as the hypothalamus, medial thalamus, and mesencephalic tegmentum (Figure 6). However, a different situation was observed when we studied the brains of BSE-H–infected mice exhibiting C-like PrP^res^ features. All these mice showed a lesion pattern comparable with that in BSE-C–infected mice, in which slight differences are found only in the mesencephalic tegmentum (Figure 6). These differences consisted of moderate lesions, whereas in BSE-C–infected mice, no lesions were found in this area. These features were conserved on secondary transmissions where no remarkable differences were found when compared with primary transmissions (data not shown).

**Figure 7:**
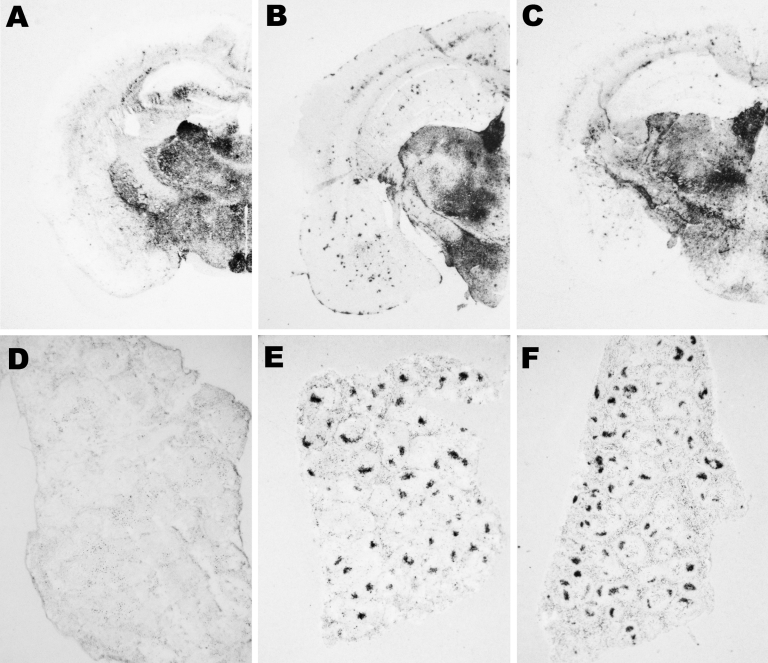
Abnormal isoform of host-encoded prion protein (PrP^Sc^) deposition patterns in brain and spleen from *Tg110* mice infected with BSE-H. A–C) Paraffin-embedded tissue (PET) blots of representative coronal sections at the level of the hippocampus from *Tg110* mice infected with atypical BSE-H (isolate 02-2695, first passage) showing either high-type (A) or classical-type protease-resistant prion protein (PrP^res^) phenotype (B). PET blot from *Tg110* mice infected with BSE-C (C) is included for comparison. D–F) PET blots of representative spleen sections: *Tg110* mice infected with atypical BSE-H (isolate 02-2695) showing high-type PrP^res^ in the brain were consistently scored as negative for PrP^Sc^ detection (D), whereas clear PrP^Sc^ deposits were always detected in BSE-H infected mice exhibiting BSE-C–like features (E), as in mice infected with BSE-C (F). Original magnification levels: panels A–C, ×20; panels D–F, ×6.BSE, bovine spongiform encephalopathy; BSE-H, unglycosylated PrP^res^ that is higher than BSE-C; BSE-C, classical BSE.

Moreover, PrP^Sc^ deposits were distinctly distributed after both primary and secondary transmissions when BSE-H–infected mice exhibiting C-type PrP^res^ features were compared with those with H-type PrP^res^, as assessed by paraffin-embedded tissue blot on brain coronal sections (Figure 7). However, the PrP^Sc^ deposition patterns were clearly similar when these mice were compared with those infected with BSE-C, after both primary and secondary transmission.

Transmission experiments (*27*) showed that, contrary to BSE-C, BSE-H is poorly lymphotropic in mouse models. The comparative study of PrP^Sc^ accumulation in spleen from our *Tg110* mice infected with both agents showed that BSE-H infected mice exhibiting H-type PrP^res^ in their brain were consistently scored as negative for PrP^Sc^ detection by paraffin-embedded tissue blot. In contrast, clear PrP^Sc^ deposits were always detected in BSE-H–infected mice exhibiting C-like features, as in mice infected with BSE-C (Figure 7). Similar results were obtained after secondary transmissions.

## Discussion

We studied the behavior and stability of the atypical BSE-H during propagation into a bovine PrP background, thus in the absence of a species barrier. We used *Tg110* mice (*29**,**36*) because they express a PrP^C^ homologous to that of the donors, thus providing a relevant context for comparing atypical BSE-H and epizootic BSE-C isolates.

Our results showed that all BSE-H isolates induced a typical neurologic disease on primary transmission, with a 100% attack rate and survival times similar to those produced by several BSE-C isolates in this mouse line (*29**,**36*) (Figure 1). The longer survival times for some mice infected with BSE-H isolates could reflect a lower infectivity of this isolate consistent with the reduction of survival time observed on subpassages, approaching that for BSE-C or BSE-H isolates of presumably higher titer (i.e., producing no substantial reduction of survival time on subpassage). These results are also consistent with another comparative study of BSE-H and BSE-C transmissions in a different bovine PrP mouse line (*27*). These data suggest that atypical BSE-H and BSE-C agents have similar transmission features into *Tg110* mice.

Although all BSE-H–inoculated mice showed homogeneous survival times, a phenotypic divergence was observed in a few animals infected with 2 of the BSE-H isolates. Surprisingly, these few mice showed phenotypic features clearly distinct from those in most of the BSE-H–infected mice but similar to those of BSE-C propagated onto the same mice, according to various criteria. First, a PrP^res^ profile indistinguishable from that produced by BSE-C agent in these mice but clearly distinct from that of BSE-H in cattle, in terms of 1) apparent molecular mass of PrP^res^, 2) PrP^res^ glycosylation pattern, 3) immunoreactivity with 12B2 mAb, and 4) pattern of labeling with Saf84 antibody. Second, the vacuolation profile essentially overlapped that in mice infected with BSE-C, with slight differences only in the mesencephalic tegmentum area. Third, the spatial distribution of PrP^res^ in the brain was clearly similar to that of mice infected with BSE-C. Fourth, PrP^Sc^ was consistently detected in the spleen, similar to mice infected with BSE-C. These similarities with BSE-C were fully retained after a second passage by using brain homogenate from mice with C-like features, whereas a BSE-H strain phenotype was maintained in mice inoculated with mouse brains homogenates containing H-type PrP^res^.

However, C-like features emerged in only 2 of the 5 isolates tested. Because only a low proportion of the mice inoculated with these 2 isolates exhibited these novel features (3/12 and 2/10, respectively), the lack of such observation in the other 3 isolates, and in 2 other independent studies of 3 BSE-H isolates in different bovine transgenic mouse lines (*27*), could be due to the low number of inoculated mice (6 per isolate), which could be statistically insufficient for such an event. No variability was ever observed in the PrP^res^ profiles of >100 *Tg110* mice inoculated with 4 different BSE-C isolates (*29**,**36*) (Figure 1). However, a divergent evolution of the BSE agent has been reported after trans-species transmission in both wild-type (*11*) and human PrP transgenic mice (*12**,**39*,*40*).

Although further studies are required to clarify the mechanisms associated with the emergence of distinct phenotypes among individual mice, several factors would be expected to influence the probability of detecting such a variant through mouse bioassay. These factors are 1) amount or regions of cattle brain tissue taken for inoculum preparation, 2) physicochemical treatment during inoculum preparation (e.g., temperature, homogenization buffer), 3) the precise site of mouse inoculation, 4) the infectious titer of the inoculum, and 5) others unknown mouse factor affecting prion propagation and disease evolution. Because samples used in this study were prepared from the same region (brainstem) following the same precise protocol and under identical conditions, differences in inoculum preparation and conditions are unlikely. However, the possibility that the observations might be influenced by the precise neuroanatomic origin of the inoculated bovine brainstem homogenate or by other mouse bioassay–related factors cannot be excluded.

The possible cross-contamination of the BSE-H isolates material (02-2695 and 45 from 2 laboratories in different countries) by a BSE-C infectious source was judged highly improbable for several reasons. These reasons are 1) the strict biosafety procedures followed for sample collection, preparation of the inocula, inoculation scheme, and care of mice; 2) the absence of C-type PrP^Sc^ in the BSE-H inocula used for transmissions as deduced by Western blot analysis; and 3) 2 independent transmission experiments, involving separate batches of both incriminated isolates, all produced consistent results.

Together, these observations support 2 possible hypotheses. First, a minor strain component might be present in BSE-H isolates that could emerge on subsequent transmission in *Tg110* mice. Second, a new strain component has been generated during propagation of BSE-H agent in *Tg110*. In both instances, emergence of the new strain, either in the original cattle or during propagation in *Tg110* mice, could be promoted by specific propagation conditions or by physicochemical treatment of the inoculum. In this regard, acquisition of novel properties by a sporadic cattle transmissible spongiform encephalopathy agent by a physicochemical treatment, such as that applied to carcass-derived products, has been invoked as a possible origin for the BSE epidemic (*7*).

Contrary to BSE-H, the atypical BSE-L agent retained unique and distinct phenotypic features, compared with BSE-C agent, on transmission to both bovine and human PrP transgenic mice (*26**–**28*). This agent, however, acquired phenotypic traits intriguingly similar to those of the BSE agent during trans-species transmission in either transgenic mice expressing ovine PrP (*28*) or inbred mouse lines. On the basis of these observations, the BSE-C agent already has been speculated to have originated from atypical BSE-L after conversion in an intermediate host such as a sheep. However, the capacity of these BSE-L–derived agents to retain BSE phenotypic traits after reinoculation to bovine PrP transgenic mice is a key question, remaining to be demonstrated, to show whether the observed convergence truly reflects a permanent strain shift of the BSE-L agent rather than a phenotypic convergence in an experimental model.

In contrast, our results suggest that prion strain divergence might occur on propagation of atypical BSE-H in a homologous bovine PrP context and that this strain divergence could result from a permanent strain shift of the BSE-H agent toward a C-like agent that is stable in subsequent passages. These findings emphasize the potential capacity of prion diversification during propagation, even in the absence of any species barrier, and represent an experimental demonstration of the capability of an atypical, presumably sporadic, bovine prion to acquire C-like properties during propagation in a homologous bovine PrP context.

Results in transgenic mouse models cannot be directly extrapolated to the natural host. However, our observations are consistent with the view that the BSE agent could have originated from a cattle prion, such as BSE-H, and provide new insights into the nature of the events that could have led to the appearance of this agent.
